# A Diagnostic Challenge: Post‐Transfusion Purpura Unmasked After Multiple Transfusions

**DOI:** 10.1002/ccr3.71831

**Published:** 2026-01-14

**Authors:** Jacintha Thomas, Priyal Gopalan, Tanya M. Wildes

**Affiliations:** ^1^ Department of Internal Medicine University of Nebraska Medical Center/Nebraska Medicine Omaha Nebraska USA; ^2^ Department of Internal Medicine, Division of Hematology/Oncology University of Nebraska Medical Center/Nebraska Medicine Omaha Nebraska USA

**Keywords:** critical care medicine, general medicine, hematology, immunology, pathology and laboratory medicine

## Abstract

Post‐transfusion purpura (PTP) is a rare immune‐mediated post‐transfusion reaction resulting in severe thrombocytopenia. This case presents a 52‐year‐old female with profound thrombocytopenia following multiple transfusions. PTP was not initially suspected due to her history of comorbid anti‐phospholipid antibody syndrome (APS), chemotherapy treatments, and recent heparin exposure. Her thrombocytopenia was refractory to intravenous immunoglobulin (IVIG), and she was started on plasmapheresis (PLEX) with significant improvement in platelet counts. Positive human platelet antigen (HPA)‐1a and HPA‐5b antibodies later confirmed a diagnosis of PTP. This case highlights the importance of early recognition of PTP, and treatment should not be delayed while awaiting confirmatory antibody testing.

## Key Clinical Message

- Post‐transfusion purpura must be considered when severe thrombocytopenia develops 5–10 days post‐transfusion, and therapy with intravenous immunoglobulin should be promptly initiated even before confirmatory HPA testing is available.

## 1 Background

Post‐transfusion purpura (PTP) is a rare immune‐mediated reaction characterized by severe thrombocytopenia (often less than 10,000 cells/μL) occurring 5–10 days post‐transfusion [1, 2, 3]. Patients will often have platelet‐specific antibodies which develop due to alloimmunization after prior transfusions or pregnancy [1]. PTP is commonly misdiagnosed given its rarity, low clinician familiarity, and limited access to confirmatory antibody testing. Identifying a transfusion as the trigger can be challenging given the delayed onset of thrombocytopenia post‐transfusion. PTP also bears clinical similarity to other immune‐mediated hematologic conditions, like immune thrombocytopenic purpura (ITP) or heparin induced thrombocytopenia (HIT), making it a diagnostic challenge (Table 1). A delayed diagnosis of PTP or misdiagnosis can lead to life‐threatening hemorrhage.

**TABLE 1:** Comparison of characteristics, timeline, and treatment of post‐transfusion purpura (PTP), immune thrombocytopenic purpura (ITP), and heparin‐induced thrombocytopenia (HIT).

	PTP	HIT	ITP
Typical onset	5–10 days after transfusion	5–10 days after heparin exposure (or sooner with prior heparin exposure)	Sudden or gradual (may follow an infection)
Mechanism	Alloantibody formation against both transfused platelets and the patient's platelets (commonly anti‐HPA1a)	Antibody formation against heparin‐platelet factor 4 (PF4) complexes– > platelet activation– > thrombosis	Autoantibody‐mediated platelet destruction
Platelet Count	Severe, often < 10,000/μL	Often > 50% drop from baseline	< 100,000/μL
Clinical Features	Sudden onset, severe thrombocytopenia; mucosal bleeding; purpura	Thrombosis (often venous); mild bleeding	Petechiae; purpura; mucosal bleeding
Key Risk Factors	Prior sensitization (pregnancy, prior transfusion)	Heparin Exposure	Viral infection, medications (antibiotics, antiepileptics, NSAIDs), underlying autoimmune disease
Diagnostic Findings	Anti‐HPA antibodies (particularly anti‐HPA1a)	Positive anti‐PF4 antibody + serotonin release assay	Isolated thrombocytopenia, otherwise normal labs
Treatment	IVIG (first‐line), PLEX and corticosteroids (for refractory cases), avoidance of platelet transfusions	Stop heparin, transition to a non‐heparin anticoagulant	IVIG and corticosteroids

## 2 Case History

A 52‐year‐old female initially presented to the emergency department (ED) at an outside hospital with fatigue, weakness, and shortness of breath 3 days after elective bilateral total knee replacements. Her hemoglobin was 5.9 g/dL and her platelet count was 142,000/μL. With no obvious source of bleeding, she received two units of packed red blood cells and was discharged home.

On day 4 post‐transfusion, she returned to the ED with severe fatigue and bilateral surgical site bleeding. Her hemoglobin was 5.2 g/dL, and her platelet count was 106,000/μL with an International Normalized Ratio (INR) of 8.0. She received two units of packed red blood cells and Vitamin K and was admitted to the outside hospital.

Her past medical history was notable for stage I ovarian clear cell carcinoma status post complete hysterectomy with bilateral salpingo‐oophorectomy followed by chemotherapy (1 year prior to the current admission) and antiphospholipid antibody syndrome (APS) complicated by a prior deep vein thrombosis, pulmonary embolism, and stroke. Her home warfarin prescription was held peri‐operatively, and enoxaparin was given until warfarin could be resumed at therapeutic levels.

On day 5 post‐transfusion, she received one unit of platelets, and hematology was consulted. With a mildly elevated total bilirubin, hemolysis labs were evaluated. She had a mildly elevated reticulocyte count, elevated lactate dehydrogenase, low haptoglobin, mildly prolonged partial thromboplastin time, elevated fibrinogen, and no evidence of schistocytes. With an INR less than 2.0, a heparin drip was started. After starting the heparin drip, her platelet count decreased from 77,000/μL to 22,000/μL.

The differential diagnosis included catastrophic APS, thrombotic thrombocytopenic purpura (TTP), ITP, and HIT, given her history of APS, autoimmune disease and heparin exposure. There was low concern for disseminated intravascular coagulation (DIC) in the setting of an elevated fibrinogen and lack of schistocytes. Given her recent exposure to enoxaparin and heparin, there was concern for HIT, and she was switched to argatroban. ADAMTS13, anti‐platelet factor 4 antibody, serotonin release assay, and direct antiglobulin tests were unremarkable, which helped rule out TTP and HIT.

On day 6 post‐transfusion, IVIG and corticosteroids were started for potential catastrophic APS. She received one unit of packed red blood cells and one unit of platelets and was transferred to our medical center for consideration of plasmapheresis (PLEX).

At our medical center, hematology was consulted, who felt her clinical presentation was not consistent with catastrophic APS given the lack of thrombosis or multiorgan dysfunction. However, given the severity of her thrombocytopenia, we continued IVIG and corticosteroids. Her platelet count initially improved with IVIG up to 68,000/μL; however, by day 4 of IVIG, her platelet count started decreasing. By day 5 of IVIG (day 10 post‐transfusion), her platelet count was 0 cells/μL.

At this point, we started rituximab and romiplostim for possible secondary ITP, and she had no improvement in platelet count. With her history of ovarian cancer and chemotherapy exposure, we performed a bone marrow biopsy, which showed cellular bone marrow with trilineage hyperplasia with mild megakaryocytic atypia favoring a reactive etiology over myelodysplastic syndrome. Cytogenetics and fluorescence in situ hybridization testing for myeloid mutations were normal.

**FIGURE 1:**
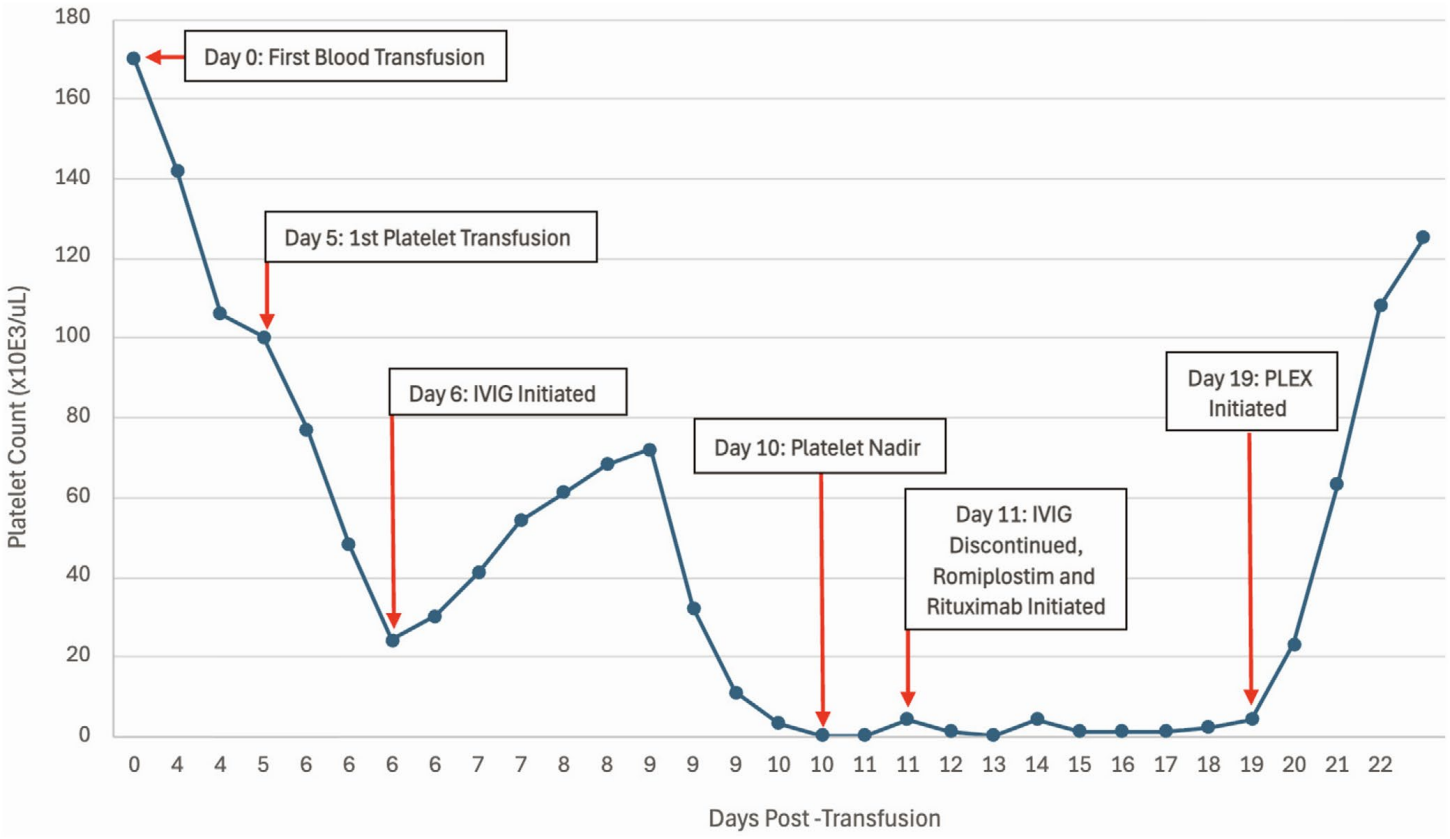
Hospital timeline from initial blood transfusion with platelet counts and interventions.

After ruling out other potential causes of thrombocytopenia, we re‐evaluated her case. PTP had not been considered earlier for multiple reasons. She had a diagnosis of underlying APS, which raised suspicion for catastrophic APS or another immune‐mediated process like ITP or TTP. She had exposure to heparin products around the same time as her initial transfusion. Given its rarity and lack of clinician familiarity, PTP was not considered until a hematologist familiar with PTP rotated onto the inpatient hematology service. At this point, it was noted that her platelet count started decreasing 6 days after the initial transfusion of blood products, and PTP was added to the differential (Figure 1). On day 19 post‐transfusion, we initiated PLEX given the lack of improvement with IVIG and corticosteroids. Following three rounds of PLEX, her platelet count increased to 125,000/μL. About 2 weeks later, our diagnosis of PTP was confirmed with the detection of human platelet antigen (HPA)‐1a and HPA‐5b antibodies.

## 3 Discussion

PTP is a rare post‐transfusion reaction characterized by severe thrombocytopenia. PTP typically develops 5–10 days following transfusion of platelets, red blood cells, or plasma, with the highest risk following platelet transfusions [1, 2, 3]. Though the true incidence of PTP is unknown, reported incidences range from 1 in 24,000 to 1 in 100,000 transfusions [3]. PTP can lead to mucosal bleeding, petechiae, ecchymoses, and intracranial hemorrhage with a mortality rate of approximately 10%–20% [3, 4, 5].

The pathophysiology of PTP involves the formation of alloantibodies directed against platelet‐specific antigens, most commonly HPA‐1a, noted in about 85% of cases [1]. Alloantibodies are produced by individuals lacking HPA‐1a on their cells, primarily individuals with the HPA‐1b/1b genotype, an uncommon genotype found in about 2% of the Caucasian population [3]. Alloantibody sensitization occurs in multiparous females exposed to platelet antigens during pregnancy and in nulliparous women and men with a history of prior transfusions [1].

When next exposed to the offending antigen during a transfusion, immune‐mediated destruction of both autologous and donor platelets results [2, 6, 7]. The mechanism of autologous platelet destruction is not fully understood. One proposed mechanism is that transfused soluble platelet antigen and autoantibodies form immune complexes, which bind nonspecifically to autologous platelets, causing indiscriminate macrophage platelet destruction [3, 5, 7]. A second potential mechanism is that soluble platelet antigens coat autologous platelets, priming them for autoantibody‐mediated destruction [3, 5]. A third possible mechanism is that autoantibodies and alloantibodies are concomitantly produced, with autoantibodies being responsible for the autologous platelet destruction [3, 5].

Diagnostic testing involves the identification of HPA platelet antibodies and platelet‐specific antigen identification, along with autologous platelet genotyping [3, 5]. Alloantibodies against HPAs will be detected in the sera via direct or indirect testing, including flow cytometry, platelet antibody bead array, and monoclonal antibody immobilization of platelet antigen [3, 5]. HPA genotyping is performed via polymerase chain reaction to distinguish it from autoantibodies and confirm HPA antibody specificity, which can be useful when considering future transfusions [3, 8].

IVIG is considered first‐line therapy for PTP, typically involving IVIG 400–500 mg/kg/day for 1–10 days or 1–2 g/kg/day for 2–5 days [3, 5, 9]. Nearly 85% of patients respond to IVIG within 2–3 days [10]. Corticosteroids have been used, though their effectiveness is not well defined [11]. Unlike typical cases of PTP, our patient's thrombocytopenia was refractory to IVIG. Prior to the widespread use of IVIG for autoimmune processes, PLEX was used to rapidly eliminate circulating platelet alloantibodies in PTP [12]. Though PLEX is more invasive and resource‐intensive than IVIG, it should be considered in cases of PTP refractory to IVIG or cases presenting with significant hemorrhaging [12, 13]. Our patient's platelet count improved exponentially after only 3 rounds of PLEX and continued to slowly improve after discontinuing PLEX. Though direct comparative data for PLEX versus IVIG in cases of PTP are limited, this case adds to the literature by demonstrating the utility of PLEX in a case of PTP refractory to IVIG. Though recurrence of PTP appears to be uncommon, HPA‐compatible blood products can be used to mitigate recurrence with future transfusions [3].

There are many barriers to making a diagnosis of PTP. First, PTP is clinically similar to other causes of autoimmune thrombocytopenia, like ITP and HIT. Our patient had underlying APS and recent heparin exposure raising suspicion for both ITP and HIT. One key distinguishing feature of PTP is the severity of the thrombocytopenia. A platelet count of 0/μL is highly characteristic of PTP and would be uncommon in other etiologies of autoimmune thrombocytopenia (Table 1).

A second barrier is that PTP is the prolonged length of time between transfusion and thrombocytopenia onset. Our patient did not reach her platelet nadir until day 10 post‐transfusion. This time frame can delay suspicion and lead to misattribution of the thrombocytopenia to other causes, including a delayed hemolytic transfusion reaction.

A third challenge includes serological testing. The identification of platelet‐specific alloantibodies is crucial for diagnosis; however, serum samples require a specialized laboratory, and results were not available to us for 2 weeks. Lastly, as PTP is a rare condition, many providers may be unaware of the condition, and those who are aware may have a lower index of suspicion given its infrequency.

## 4 Conclusion

This case report aims to increase awareness of PTP, which is a rare, immune‐mediated reaction resulting in severe thrombocytopenia that occurs after a transfusion of blood products. The cause of thrombocytopenia can often be misattributed to other, more well‐known conditions, like ITP or HIT. Two key components of PTP to recognize are the onset of thrombocytopenia occurring within 5–10 days of blood product transfusion and the severity of the thrombocytopenia, with platelet counts often less than 10,000/μL.

Though HPA‐1a and HPA‐5b antibodies may confirm a diagnosis of PTP, these tests require specialized laboratories and take time to yield results. In patients with unexplained severe thrombocytopenia occurring shortly after transfusion, clinicians should avoid delays in considering PTP. IVIG should be initiated quickly, even before confirmatory antibody testing results are available.

Diagnosing our patient with PTP was diagnostically challenging given the confounders of her APS, recent heparin use, and prior chemotherapy exposure, leading to a delay in diagnosis. Our case adds to the literature by demonstrating the utility of PLEX in patients whose thrombocytopenia is refractory to first‐line IVIG therapy.

## Author Contributions

**Jacintha Thomas:** conceptualization, methodology, project administration, writing – original draft, writing – review and editing. **Priyal Gopalan:** conceptualization, supervision, writing – review and editing. **Tanya M. Wildes:** conceptualization, supervision, writing – review and editing.

## Funding

The authors have nothing report.

## Consent

Written informed patient consent was obtained for publication of the case details.

## Conflicts of Interest

The authors declare no conflicts of interest.

## Data Availability

Data sharing not applicable to this article as no datasets were generated or analyzed during the current study.

## References

[1] A. Ziman , E. Klapper , S. Pepkowitz , R. Smith , G. Garratty , and D. Goldfinger , “A Second Case of Post‐Transfusion Purpura Caused by HPA‐5a Antibodies: Successful Treatment With Intravenous Immunoglobulin,” Vox Sanguinis 83, no. 2 (2002): 165–166. 12201847 10.1046/j.1423-0410.2002.00207.x

[2] K. Vu and A. D. Leavitt , “Posttransfusion Purpura With Antibodies Against Human Platelet Antigen‐4a Following Checkpoint Inhibitor Therapy: A Case Report and Review of the Literature,” Transfusion 58, no. 10 (2018): 2265–2269. 30222869 10.1111/trf.14824

[3] J. Hawkins , R. H. Aster , and B. R. Curtis , “Post‐Transfusion Purpura: Current Perspectives,” Journal of Blood Medicine 10 (2019): 405–415. 31849555 10.2147/JBM.S189176 PMC6910090

[4] A. W. Loren and C. S. Abrams , “Efficacy of HPA‐1a (PlA1)‐Negative Platelets in a Patient With Post‐Transfusion Purpura,” American Journal of Hematology 76, no. 3 (2004): 258–262. 15224362 10.1002/ajh.20093

[5] L. Owczarzak , T. Alrifai , S. Jain , and I. Dehghan‐Paz , “Uncommon Presentation of Post‐Transfusion Purpura in an Elderly Male: A Case Report and Unique Alloantibody Identification,” American Journal of Case Reports 25 (2024): e942949. 38978279 10.12659/AJCR.942949 PMC11318730

[6] T. Kickler , P. Ness , J. Herman , and W. Bell , “Studies on the Pathophysiology of Posttransfusion Purpura,” Blood 68, no. 2 (1986): 347–350. 3524706

[7] C. Woelke , P. Eichler , G. Washington , and B. K. Flesch , “Post‐Transfusion Purpura in a Patient With HPA‐1a and GPIa/IIa Antibodies,” Transfusion Medicine 16, no. 1 (2006): 69–72. 16480442 10.1111/j.1365-3148.2005.00633.x

[8] M. Menis , R. A. Forshee , S. A. Anderson , et al., “Posttransfusion Purpura Occurrence and Potential Risk Factors Among the Inpatient US Elderly, as Recorded in Large Medicare Databases During 2011 Through 2012,” Transfusion 55, no. 2 (2015): 284–295. 25065878 10.1111/trf.12782

[9] C. Mueller‐Eckhardt , E. Küenzlen , D. Thilo‐Körner , and H. Pralle , “High‐Dose Intravenous Immunoglobulin for Post‐Transfusion Purpura,” New England Journal of Medicine 308, no. 5 (1983): 287. 10.1056/NEJM198302033080526 6681546

[10] B. J. Hunt , “Bleeding and Coagulopathies in Critical Care,” New England Journal of Medicine 370, no. 9 (2014): 847–859. 24571757 10.1056/NEJMra1208626

[11] L. J. Weisberg and C. A. Linker , “Prednisone Therapy of Post‐Transfusion Purpura,” Annals of Internal Medicine 100, no. 1 (1984): 76–77. 6537880 10.7326/0003-4819-100-1-76

[12] P. L. Cimo and R. H. Aster , “Post‐Transfusion Purpura: Successful Treatment by Exchange Transfusion,” New England Journal of Medicine 287, no. 6 (1972): 290–292. 5038955 10.1056/NEJM197208102870608

[13] B. Laursen , N. Morling , J. Rosenkvist , H. Sørensen , and S. Thyme , “Post‐Transfusion Purpura Treated With Plasma Exchange by Haemonetics Cell Separator. A Case Report,” Acta Medica Scandinavica 203, no. 6 (1978): 539–543. 566507 10.1111/j.0954-6820.1978.tb14922.x

